# The National Hospital

**Published:** 1901-07-13

**Authors:** 


### The National Hospital.

A general meeting of the governors of the National Hospital for the Paralysed and Epileptic was held on the 6th instant, for the purpose of considering the report of the committee of inquiry. Only three members of the Board of Management were present—Mr. Pearman, who was voted to the chair, Mr. Russell, who left as soon as the first resolution had been carried, and Mr. Campbell. A special appeal for attendance had been made to the governors, in a letter addressed to the *Times* by Mr. Melvill Green, and this appeal had been enforced by a prominent editorial paragraph in the *Pall Mall Gazette*; but, if the members of the staff, of the Board of Management, and of the original committee of selection were omitted, the rest of the governors who came might perhaps have been counted on the fingers of two hands. This was partly due, no doubt, to the circumstance that the board had adhered to their old policy of keeping their constituents as much at arm's length as possible, and had fixed the meeting for twelve o'clock on a Saturday, so that it might come into direct conflict alike with the luncheon hour and with the prevailing tendency to a "week-end" holiday. Even when allowance was made for the obstacles thus artificially created, the thinness of attendance afforded a lamentable proof of the real indifference of the public towards the institutions which they support. The adoption of the report of the committee of inquiry was moved by Mr. Melvill Green, who pointed out that the acceptance of his resolution pledged the governors irrevocably to the abolition of the office of "Secretary-Director," to the addition to the Board of Management of two members of the medical staff to be selected by their colleagues, and to a complete reconstruction of the rules under which the hospital has hitherto been governed. These results had been accepted as inevitable by the Board of Management in a conference with the committee prior to the meeting, and the resolution, having been seconded by Sir James Crichton Browne, was carried *nem. con.* Mr. Green then moved that a committee should be appointed to represent the governors in making such arrangements as may be necessary for dealing with all matters consequential upon, or arising out of, the adoption of the previous resolution. He explained that such a committee would report its recommendations and acts to a final meeting of governors, but that this could not be held before October, and that, in the meanwhile, certain decisions must be arrived at, among which he mentioned the settlement of a pension upon Mr. Burford Rawlings on the abolition of his office as director, and on his resignation of the secretaryship, which he had filled with great efficiency for five and thirty years. A third resolution provided that the committee should consist of nine persons, not necessarily governors, of whom four should be chosen by the original committee of selection, three by the Board of Management, and two by the staff. This would have been opposed, had it not been privately announced that the committee of selection would appoint Mr. Melvill Green himself, Mr. Timothy Holmes, Mr. Danvers Power, and Mr. Wigan as their nominees, an arrangement with which the staff was content. Sir Felix Semon and Dr. Ormerod were then appointed by the staff, Messrs. Pearman, Russell, and Campbell by the board, and the business was brought to a conclusion.

We fear it cannot be expected that the arrangements thus temporarily made will work without friction, or that the victory of sound principles, which the report of the committee of inquiry appeared to indicate, can yet be looked upon as gained. Contrary to all reasonable expectation, the members of the Board of Management have retained their seats; and no one who is conversant with their past system of government can feel any confidence that they will cordially accept the altered conditions under which they will be placed. It is far more likely that they will endeavour to escape from these conditions by devious ways, or to neutralise them by cunningly devised intrigue. The apathy of the governors, as displayed by the scanty attendance at Saturday's meeting, will afford only too much encouragement to the hope that by lying low for a time, and waiting upon events, something like the original state of affairs may be restored; and the new medical members of the board will, we fear, enter upon their duties with the feeling that they have to contend against watchful adversaries, rather than to co-operate heartily with friendly colleagues.

